# Dynamics in treatment response and disease progression of metastatic colorectal cancer (mCRC) patients with focus on BRAF status and primary tumor location: analysis of untreated RAS-wild-type mCRC patients receiving FOLFOXIRI either with or without panitumumab in the VOLFI trial (AIO KRK0109)

**DOI:** 10.1007/s00432-020-03257-z

**Published:** 2020-05-24

**Authors:** A. Kurreck, M. Geissler, U. M. Martens, J. Riera-Knorrenschild, J. Greeve, A. Florschütz, S. Wessendorf, T. Ettrich, S. Kanzler, D. Nörenberg, M. Seidensticker, S. Held, P. Buechner-Steudel, J. Atzpodien, V. Heinemann, S. Stintzing, T. Seufferlein, A. Tannapfel, A. C. Reinacher-Schick, D. P. Modest

**Affiliations:** 1 Department of Hematology, Oncology, and Tumor Immunology (CVK/CCM), Charité University Medicine Berlin, Augustenburger Platz 1, 13353 Berlin, Germany; 2 Klinikum Esslingen, Esslingen, Germany; 3 Klinik für Innere Medizin III, SLK-Kliniken Heilbronn, Heilbronn, Germany; 4 Universitätsklinik Marburg, Marburg, Germany; 5 St. Vincenz-Krankenhaus Paderborn, Paderborn, Germany; 6 Stadtisches Klinikum Dessau, Dessau, Germany; 7 Universitätsklinikum Ulm, Ulm, Germany; 8 Leopoldina Krankenhaus, Schweinfurt, Germany; 9 Medical Faculty Mannheim, Institute of Clinical Radiology and Nuclear Medicine, University Medical Center Mannheim, Heidelberg University, Mannheim, Germany; 10 Klinik Und Poliklinik für Radiologie, LMU Klinikum, München, Germany; 11 ClinAssess, Leverkusen, Germany; 12 Universitätsklinikum Halle (Saale), Halle, Germany; 13 Franziskus-Hospital Harderberg, Georgsmarienhütte, Germany; 14 Department of Medicine III and Comprehensive Cancer Center, University Hospital Munich (LMU), Munich, Germany; 15 German Cancer Consortium (DKTK), German Cancer Research Center, Heidelberg, Germany; 16 Institute of Pathology, Ruhr-University Bochum, Bochum, Germany; 17 Department of Hematology, Oncology and Palliative Care, St. Josef Hospital, Ruhr-University Bochum, Bochum, Germany

**Keywords:** Metastatic colorectal cancer, Disease dynamics, Depth of response, Early tumor shrinkage, BRAF, Primary tumor site, Combination chemotherapy

## Abstract

**Purpose:**

In mCRC, disease dynamics may play a critical role in the understanding of long-term outcome. We evaluated depth of response (DpR), time to DpR, and post-DpR survival as relevant endpoints.

**Methods:**

We analyzed DpR by central review of computer tomography images (change from baseline to smallest tumor diameter), early tumor shrinkage (≥ 20% reduction in tumor diameter at first reassessment), time to DpR (study randomization to DpR-image), post-DpR progression-free survival (pPFS = DpR-image to tumor progression or death), and post-DpR overall survival (pOS = DpR-image to death) with special focus on BRAF status in 66 patients and primary tumor site in 86 patients treated within the VOLFI-trial, respectively.

**Results:**

BRAF wild-type (BRAF-WT) compared to BRAF mutant (BRAF-MT) patients had greater DpR (− 57.6% vs. − 40.8%, *p* = 0.013) with a comparable time to DpR [4.0 (95% CI 3.1–4.4) vs. 3.9 (95% CI 2.5–5.5) months; *p* = 0.8852]. pPFS was 6.5 (95% CI 4.9–8.0) versus 2.6 (95% CI 1.2–4.0) months in favor of BRAF-WT patients (HR 0.24 (95% CI 0.11–0.53); *p* < 0.001). This transferred into a significant difference in pOS [33.6 (95% CI 26.0–41.3) vs. 5.4 (95% CI 5.0–5.9) months; HR 0.27 (95% CI 0.13–0.55); *p* < 0.001]. Similar observations were made for patients stratified for primary tumor site.

**Conclusions:**

BRAF-MT patients derive a less profound treatment response compared to BRAF-WT patients. The difference in outcome according to BRAF status is evident after achievement of DpR with BRAF-MT patients hardly deriving any further disease control beyond DpR. Our observations hint towards an aggressive tumor evolution in BRAF-MT tumors, which may already be molecularly detectable at the time of DpR.

## Introduction

Colorectal cancer (CRC) represents one of the most commonly diagnosed cancers in the Western World (Boyle and Langman 2000).

Within the entity of metastatic colorectal cancer (mCRC), there are substantial differences in tumor biology (i.e., *RAS*-mutant tumors, *BRAF*-mutant tumors, tumors with microsatellite-instability or Her2/neu expression) that determine treatment choices and outcome (Douillard et al. 2013; Heinemann et al. 2014; Kopetz et al. 2019; Overman et al. 2018; Sartore-Bianchi et al. 2016; Van Cutsem et al. 2016). Additionally, primary tumor location has a functional role as a biomarker with impact on prognosis and efficacy of EGFR-antibody-based therapy (Arnold et al. 2017; Holch et al. 2017; Modest et al. 2014). Besides molecularly defined subgroups, different tumor biology can also be characterized by dynamics in early treatment response, usually described as early tumor shrinkage (ETS) or depth of response (DpR)-parameters which are also known to affect long-term survival (Cremolini et al. 2015a; Douillard et al. 2015; Giessen et al. 2013; Heinemann et al. 2015; Modest et al. 2013; Modest et al. 2017; Piessevaux et al. 2009; Piessevaux et al. 2013; Stintzing et al. 2016). The association of ETS and DpR with long-term survival is interpreted as an early identification of treatment-sensitive tumors, therefore, maybe also providing a valuable tool in the context of secondary resection of metastases (Modest et al. 2020). To the best of our knowledge, the dynamics and clinical course of mCRC beyond these early endpoints (i.e., ETS and in particular DpR) have been explored less rather in terms of a definition of endpoints related to progression-free survival (Chibaudel et al. 2011). In particular, (progression-free) survival outcomes after DpR that might reflect the ability of a disease to overcome a certain treatment are potentially representing an interesting assessment to identify tumors with biological aggressiveness despite initial disease control.

The randomized, open-label phase II VOLFI study (AIO KRK0109) evaluated the efficacy and safety of adding the EGFR-inhibitor panitumumab to triplet chemotherapy with fluorouracil/folinic acid, oxaliplatin, and irinotecan (FOLFOXIRI) in untreated RAS-wild-type mCRC patients (Modest et al. 2019b). A central review allowing for DpR and ETS calculation was available for the trial, also providing an opportunity to investigate on tumor dynamics beyond DpR.

The aim of our study is to evaluate to which extent established (DpR, ETS) and exploratory endpoints related to best response assessment (time to DpR from randomization as well as progression-free and overall survival from time of DpR) can be used to characterize patients with known differences in tumor biology within the trial. In this regard, we evaluated the study cohort of patients according to BRAF mutational status and primary tumor location as established prognostic (and predictive) markers in mCRC. Furthermore, we analyzed patterns of progression (new lesions, progression of target lesions, etc.) and their association with the aforementioned prognostic markers in the trial.

## Methods

We performed an exploratory analysis of the randomized, open-label phase II VOLFI study (AIO KRK0109) that evaluated the efficacy and safety of adding panitumumab to triplet chemotherapy with fluorouracil/folinic acid, oxaliplatin, and irinotecan (FOLFOXIRI) in untreated RAS-wild-type mCRC patients.

A total of 96 patients were enrolled in this study to either receive chemotherapy according FOLFOXIRI protocol in the control arm or modified FOLFOXIRI (mFOLFOXIRI) plus panitumumab in the experimental arm.

The study was conducted in accordance with the ethical standards of the institutional and national research committee and with the 1964 Helsinki Declaration and its later amendments or comparable ethical standards. The ethical approval of the underlying VOLFI-trial was provided by the ethics committee of the Landesärztekammer Baden-Württemberg in Germany. For further information, please refer to the published trial data and original publication (ClinicalTrials.gov, NCT01328171) (Modest et al. 2019b).

### Patients

A clinical database was established including the following information for each patient: treatment arm, age, sex, performance status according to Eastern Cooperative Oncology Group (ECOG), tumor characteristics (primary tumor location, onset of metastases, metastatic sites), laboratory parameters (carcinoembryonic antigen, lactate dehydrogenase), molecular characteristics (*RAS* and *BRAF* status), prior antitumor treatment (adjuvant chemotherapy, surgery), and survival parameters (PFS, OS). Study patients were stratified for *BRAF* status and primary tumor location, respectively. Primary tumor location was considered “left” if the primary was located in the splenic flexure or distal, whereas the “right”-sided primaries were located proximal of the splenic flexure.

### Treatment

Treatment details are described previously (Modest et al. 2019b). Briefly, patients treated within the experimental arm received FOLFOXIRI and panitumumab in the final dosing cohort as follows: irinotecan 150 mg/m^2^, oxaliplatin 85 mg/m^2^, folinic acid 200 mg/m^2^, fluorouracil 3000 mg/m^2^ within 48 h plus panitumumab 6 mg per kilogram of body weight. Patients receiving FOLFOXIRI without panitumumab in the control arm were treated as follows: irinotecan 165 mg/m^2^, oxaliplatin 85 mg/m^2^, folinic acid 200 mg/m^2^, and fluorouracil 3200 mg/m^2^ within 48 h. Therapy in both treatment arms was repeated every 2 weeks until progression, occurrence of unacceptable toxicity, achievement of tumor resectability or up to a maximum of 12 treatment cycles.

### Disease and toxicity assessments

Tumor assessments were performed using computed tomography (CT) or magnetic resonance imaging (MRI) and subsequently analyzed according to Response Evaluation Criteria in Solid Tumors (RECIST version 1.1). After initial assessment within 21 days prior to the study start, reassessments were performed every four cycles of treatment. Afterwards, assessments were carried out until the patient’s death or up to a maximum of 5 years. Adverse events were documented according to The National Cancer Institute Common Terminology Criteria for Adverse Events (version 4.0).

### Survival endpoints and parameters indicating dynamics in treatment response and disease progression

Progression-free survival (PFS) was defined as time from study randomization to tumor progression or death from any cause. Overall survival (OS) was measured from randomization to death from any cause. Patients without progression or death were censored at the last day of follow-up.

**Fig. 1:**
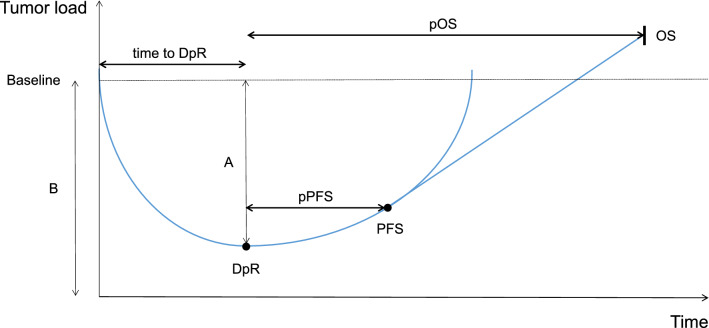
Simplified theoretical model of parameters indicating dynamics in treatment response. *DpR* depth of response, *pPFS* post-DpR progression-free survival, *pOS* post-DpR overall survival, *DpR* A/B × 100%

We evaluated the depth of response (change from baseline to smallest tumor diameter), early tumor shrinkage (> 20% reduction in tumor diameter at first reassessment) as described in the previous publication, time to DpR (study randomization to DpR-image), post-DpR PFS (pPFS = DpR image to tumor progression or death from any cause), and post-DpR OS (pOS = DpR image to death from any cause) by central review of computed tomography images (Modest et al. 2019b). Figure 1 contains a simplified model of the above-mentioned parameters.

In case of radiological disease progression in accordance with RECIST version 1.1, we described progression according to the localization of disease progression: new lesion/s, progression of target lesion/s, progression of non-target lesion/s, new lesion/s and progression of target lesion/s, new lesion/s and progression of non-target lesion/s, progression of target and non-target lesions, and new lesion/s and progression of target and non-target lesions.

### Statistical analysis

All statistical analyses were performed using SPSS version 25.0 software (IBM Corporation, Amonk, NY, USA). In univariate analyses, Chi-square tests were used to evaluate whether there is an association between BRAF mutational status or primary tumor location and the aforementioned parameters indicating dynamics in treatment response and disease progression. The two-sided significance level was set to 0.05 with a 95% confidence interval. Survival was expressed by Kaplan–Meier method and compared by log-rank testing as well as Cox regression model.

## Results

### Patient and tumor characteristics

**Fig. 2:**
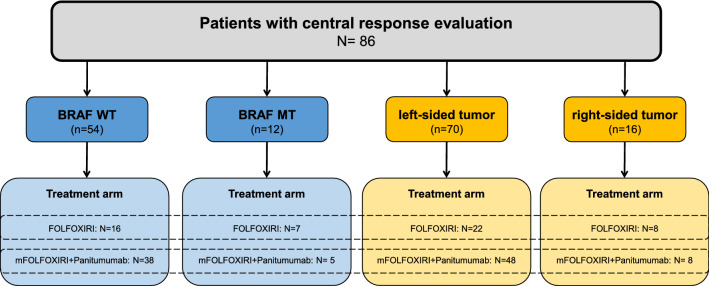
Consort diagram of study population. Information on BRAF mutational status were available for 66 patients with central response evaluation

Out of 96 patients treated within the VOLFI-trial, *BRAF* mutational status was available for 76 patients. Of those, 66 patients had been included in the central radiological review. Out of the 66 patients, 54 presented with *BRAF*-WT and 12 with *BRAF*-MT mCRC. For all patients in the full analysis set (*N* = 96), information on localization of primary tumor were available and of those, 86 patients (70 patients with left-sided colorectal cancer and 16 patients with right-sided tumor) were included in the central response evaluation. A consort diagram illustrating the study population is shown in Fig. 2. Due to overlappings between patients analyzed for BRAF mutational status and patients analyzed for primary tumor localization, the sum of the digits indicated in Fig. 2 is higher than the total number of patients with central response evaluation (*N* = 86).

Please also refer to Table 1 for baseline tumor and patient characteristics depending on *BRAF* mutational status and primary tumor location.

**Table 1:** Baseline tumor and patient characteristics

	BRAF		Primary tumor site	
	Mutant (*N* = 12)	Wild type (*N* = 54)	Left-sided (*N* = 70)	Right-sided (*N* = 16)
Median age (range), years	60 (32–71)	59.5 (33–76)	58 (31–77)	62 (32–72)
Sex				
Male	6 (50.0%)	38 (70.4%)	50 (71.4%)	9 (56.3%)
Female	6 (50.0%)	16 (29.6%)	20 (28.6%)	7 (43.8%)
Treatment arm				
FOLFOXIRI	7 (58.3%)	16 (29.6%)	22 (31.4%)	8 (50.0%)
mFOLFOXIRI + panitumumab	5 (41.7%)	38 (70.4%)	48 (68.6%)	8 (50.0%)
BRAF mutational status				
Wild type			48 (68.6%)	6 (37.5%)
Mutant			6 (8.6%)	6 (37.5%)
Unknown			16 (22.9%)	4 (25.0%)
Primary tumor location				
Left sided	6 (50.0%)	43 (79.6%)		
Right sided	6 (50.0%)	11 (20.4%)		
ECOG performance status				
0	6 (50.0%)	35 (64.8%)	45 (64.3%)	8 (50.0%)
1	6 (50.0%)	18 (33.3%)	23 (32.9%)	8 (50.0%)
2	0 (0.0%)	1 (1.9%)	2 (2.9%)	0 (0.0%)
Carcinoembryonic antigen				
≤ 50 ng/ml	7 (58.3%)	24 (44.4%)	33 (47.1%)	8 (50.0%)
> 50 ng/ml	5 (41.7%)	28 (51.9%)	35 (50.0%)	8 (50.0%)
Unknown	0 (0.0%)	2 (3.7%)	2 (2.9%)	0 (0.0%)
Lactate dehydrogenase				
≤ 250 U/l	6 (50.0%)	23 (42.6%)	31 (44.3%)	8 (50.0%)
> 250 U/l	6 (50.0%)	28 (51.9%)	36 (51.4%)	8 (50.0%)
Unknown	0 (0.0%)	3 (5.6%)	3 (4.3%)	0 (0.0%)
Tumor-related symptoms				
Yes	8 (66.7%)	24 (44.4%)	32 (45.7%)	9 (43.8%)
No	4 (33.3%)	29 (53.7%)	37 (52.9%)	7 (56.3%)
Unknown	0 (0.0%)	1 (1.9%)	1 (1.4%)	0 (0.0%)
Metastatic sites				
Liver	10 (83.3%)	50 (92.6%)	65 (92.9%)	12 (75.0%)
Lung	2 (16.7%)	13 (24.1%)	18 (25.7%)	5 (31.3%)
Peritoneum	4 (33.3%)	2 (3.7%)	3 (4.3%)	4 (25.0%)
Distant lymph node/s	1 (8.3%)	11 (20.4%)	15 (21.4%)	3 (18.8%)
Primary surgery				
Yes	5 (41.7%)	27 (50.0)	34 (48.6%)	10 (62.5%)
No	6 (50.0%)	26 (48.1%)	35 (50.0%)	5 (31.3%)
Unknown	1 (8.3%)	1 (1.9%)	1 (1.4%)	1 (6.3%)
Adjuvant chemotherapy				
Yes	0 (0.0%)	6 (11.1%)	8 (11.4%)	0 (0.0%)
No	11 (91.7%)	47 (87.0%)	61 (87.1%)	15 (93.8%)
Unknown	1 (8.3%)	1 (1.9%)	1 (1.4%)	1 (6.3%)

*ECOG* performance status according to Eastern Cooperative Oncology Group

### Dynamics in treatment response and disease progression according to BRAF mutational status

**Fig. 3:**
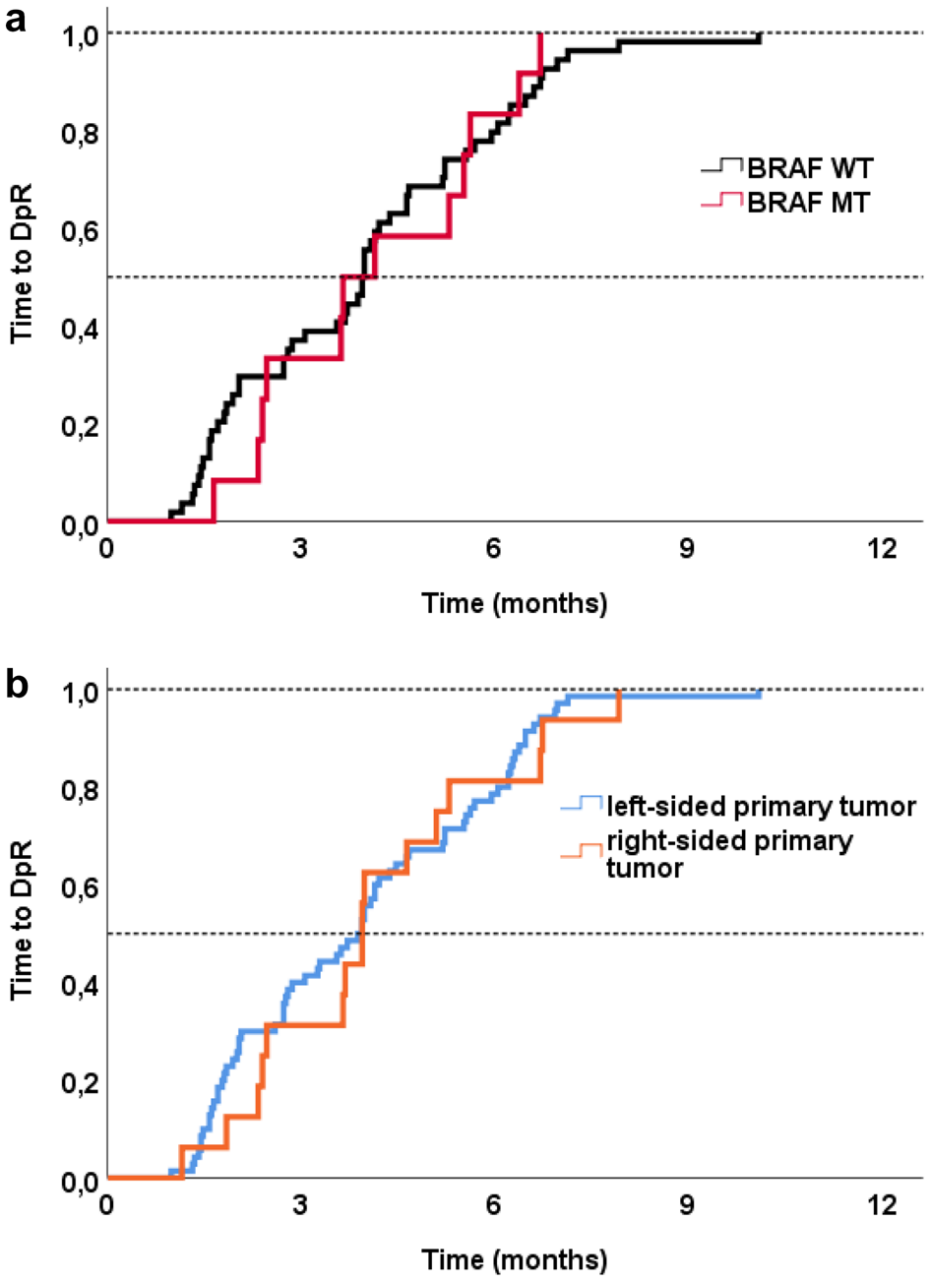
**a**, **b** Time to DpR according to BRAF mutational status and primary tumor site. *WT* wild type, *MT* mutant

*BRAF*-WT compared to *BRAF*-MT mCRC patients had greater DpR (− 57.6% vs. − 40.8%, *p* = 0.013) with a comparable time to DpR (*BRAF*-WT 4.0 months (95% CI 3.1–4.4 months) vs. *BRAF*-MT 3.9 months (95% CI 2.5–5.5 months); *p* = 0.885) as shown in Fig. 3a.

In addition, *BRAF*-WT patients achieved a higher rate of median early tumor shrinkage (ETS) at time of first radiological reassessment in comparison with *BRAF*-MT patients [85.2% (95% CI 72.9–93.4%) vs. 33.3% (95% CI 9.9–65.1%); *p* = 0.001].

**Fig. 4:**
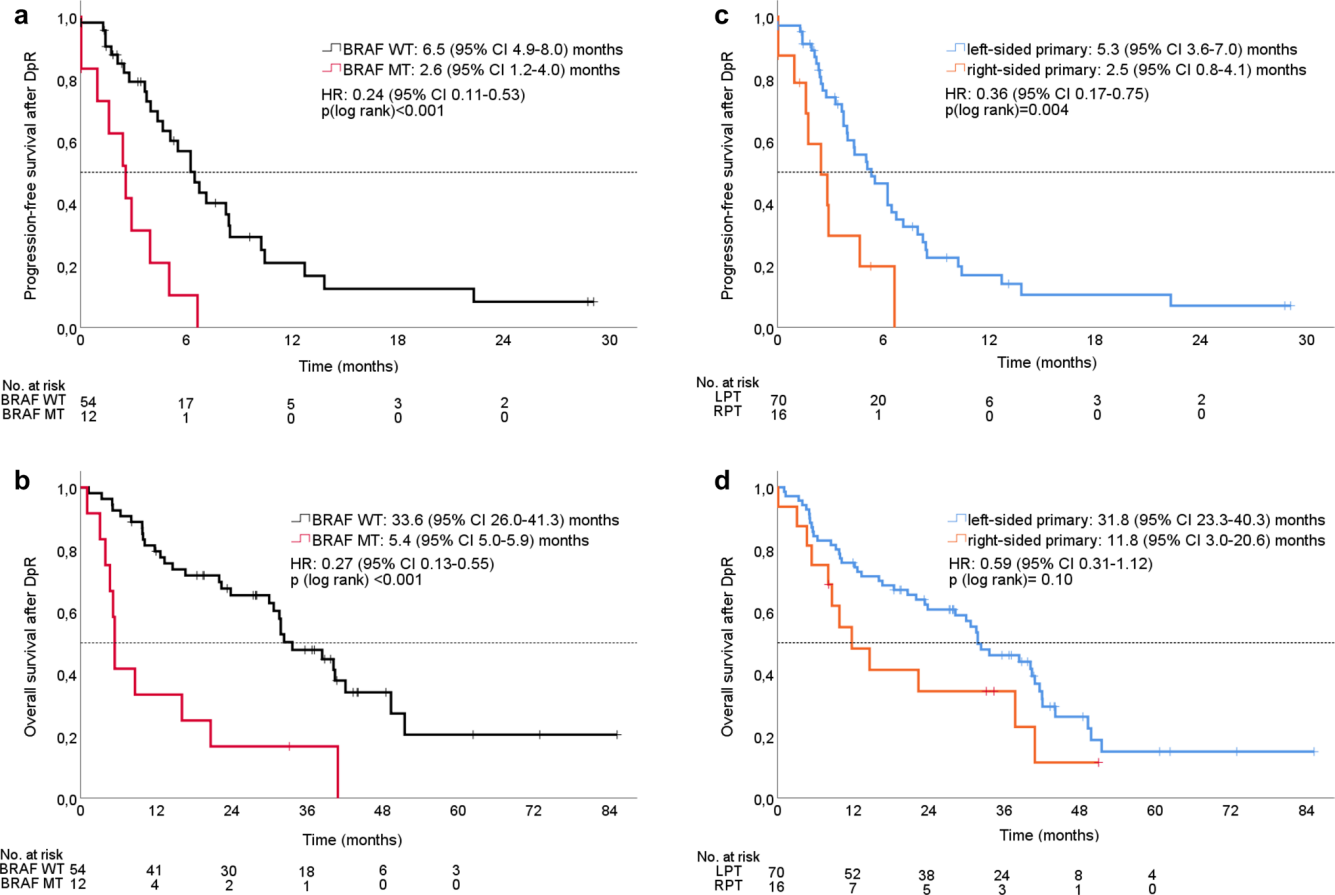
**a**–**d** Survival after depth of response (pPFS and pOS) according to BRAF mutational status and primary tumor site. **a** Post-DpR PFS depending on BRAF mutational status, **b** post-DpR OS depending on BRAF mutational status, **c** post-DpR PFS depending on primary tumor location, **d** post-DpR OS depending on primary tumor location. *WT* wild type, *MT* mutant

Post-DpR PFS (pPFS) was 6.5 months (95% CI 4.9–8.0 months) versus 2.6 months (95% CI 1.2–4.0 months) in favor of BRAF-WT patients [hazard ratio: 0.24 (95% CI 0.11–0.53); *p* < 0.001] as shown in Fig. 4a.

In accordance with pPFS, post-DpR OS (pOS) was also significantly prolonged in BRAF-WT (33.6 months, 95% CI 26.0–41.3 months) as compared to BRAF-MT patients [5.4 months (95% CI 5.0–5.9 months); hazard ratio: 0.27 (95% CI 0.13–0.55); *p* < 0.001]. Please refer to Fig. 4b.

### Dynamics in treatment response and disease progression according to primary tumor location

Patients with left-sided primary tumor achieved greater DpR compared to patients with right-sided primary tumor [− 56.8% (95% CI − 75.3 to 92.9%) vs. -30.6% (95% CI − 15.2 to 64.6%); *p* = 0.018] with a comparable time to DpR [left-sided: 3.9 months (95% CI 2.9–4.2 months) vs. right-sided: 3.9 months (95% CI 2.5–5.1 months); *p* = 0.666] as shown in Fig. 3b.

In addition, patients with left-sided tumor achieved a higher rate of median ETS at the time of first radiological reassessment compared to patients with right-sided tumor (85.7% vs. 37.5%; *p* < 0.001).

pPFS was 5.3 months (95% CI 3.6–7.0 months) versus 2.5 months (95% CI 0.8–4.1 months) in favor of patients with left-sided tumor localization [hazard ratio 0.36 (95% CI 0.17–0.75); *p* = 0.004]. Please refer to Fig. 4c.

pOS numerically favored patients with left- versus right-sided tumors, but did not reach the level of statistical significance [left-sided tumors: 31.8 months, 95% CI 23.3–40.3 months vs. right-sided tumors: 11.8 months (95% CI 3.0–20.6 months); hazard ratio: 0.59 (95% CI 0.31–1.12); *p* = 0.104] as shown in Fig. 4d.

### Disease progression patterns according to BRAF mutational status and primary tumor location

In the group of *BRAF*-WT patients and patients with left-sided primary tumor, there was a higher percentage of patients without disease progression during the observation period compared to *BRAF*-MT patients and patients with right-sided primary tumor, respectively (*BRAF*-WT vs. *BRAF*-MT: 48.1% vs. 25.0%; *p* = 0.144; left-sided vs. right-sided primary tumor: 44.3% vs. 31.3%; *p* = 0.340).

Most of the patients developed progressive disease in target lesions irrespective of *BRAF* mutational status and primary tumor location (*BRAF*-WT: 24.1%, *BRAF*-MT: 25.0%, left-sided primary tumor: 25.7%, right-sided primary tumor: 25.0%).

There was a trend towards higher frequency of disease progression in target and non-target lesions in *BRAF*-MT patients and patients with right-sided primary tumor (*BRAF*-MT vs. *BRAF*-WT: 25.0% vs. 3.7%, right-sided vs. left-sided tumor: 18.8% vs. 8.6%). Notably, the frequency of disease progression presenting with new tumor lesions was comparable in all analyzed subsets of the trial. Please refer to Table 2 for further details.

**Table 2:** Disease progression patterns according to BRAF mutational status and primary tumor location

Disease progression pattern, *N* (%)	BRAF-WT (*N* = 54)	BRAF-MT (*N* = 12)	Left-sided tumor (*N* = 70)	Right-sided tumor (*N* = 16)
New lesion/s	6 (11.1)	1 (8.3)	8 (11.4)	1 (6.3)
Progression of target lesion/s	13 (24.1)	3 (25.0)	18 (25.7)	4 (25.0)
Progression of non-target lesion/s	1 (1.9)	1 (8.3)	1 (1.4)	1 (6.3)
New lesion/s and progression of target lesion/s	3 (5.6)	0 (0.0)	4 (5.7)	0 (0.0)
New lesion/s and progression of non-target lesion/s	2 (3.7)	0 (0.0)	1 (1.4)	1 (6.3)
Progression of target and non-target lesions	2 (3.7)	3 (25.0)	6 (8.6)	3 (18.8)
New lesion/s and progression of target and non-target lesions	1 (1.9)	1 (8.3)	1 (1.4)	1 (6.3)
No progression	22 (48.1)	3 (25.0)	31 (44.3)	5 (31.3)
Progression including new lesions	11 (20.4)	2 (16.7)	14 (20.0)	3 (18.8)

*WT* wild type, *MT* mutant

## Discussion

The presence of *BRAF* mutation and/or right-sided primary tumor are associated with unfavorable survival in patients with mCRC (Clarke and Kopetz 2015; Yahagi et al. 2016). The aim of this manuscript is to use established early response assessments such as ETS and DpR as well as time-to-DpR and survival beyond DpR as novel parameters related to these endpoints to elucidate to which extent the negative prognostic impact of *BRAF* mutation and right-sided tumor localization can be characterized by these parameters.

In the VOLFI trial, both ETS and DpR were more favorable in patients with positive prognostic markers (*BRAF*-WT and left-sided primary tumor). However, time-to-DpR (maybe indicating initial treatment sensitivity) was comparable in all analyzed subgroups. Our results concerning the impact of tumor localization on early efficacy are supported by two retrospective analyses reporting higher frequencies of ETS in mCRC patients with left-sided primary tumors compared to those with right-sided tumors (Kohne et al. 2019; Peeters et al. 2018). In addition, DpR was also more pronounced in left-sided tumors in a post hoc analysis of PRIME and PEAK study (Peeters et al. 2018) while this was not evident in a smaller study cohort treated with FOLFIRI plus panitumumab in the first-line setting (Kohne et al. 2019). It should be noted that in the underlying trial and also in general there is a relevant association of *BRAF*-mutation with right-sided primary tumor localization affecting our overlapping findings (Kohne et al. 2019; Roth et al. 2010; Tran et al. 2011).

More striking than the observed disparities in ETS and DpR are substantial survival differences in our analysis occurring after DpR. In the VOLFI trial, patients with *BRAF*-MT mCRC and patients with right-sided primary tumor hardly derive any further disease control beyond DpR. The fact that DpR nearly directly precedes disease progression and, therefore, treatment failure, especially in patients with *BRAF*-MT mCRC, is alarming and could stimulate alertness in the clinical management—particularly regarding the frequency of radiological control of tumor dynamics—of these patients. Biologically, this finding suggests that mechanisms of resistance might be already ongoing despite observed tumor control or even radiological remissions. This resistance can be either interpreted as primary resistance following clonal selection of preexisting chemotherapy-resistant cells responsible for disease progression after DpR, or alternatively as rapid tumor evolution. This in turn hints to the hypothesis that *BRAF*-MT tumors are somewhat less sensitive to chemotherapy-based antitumor treatment in terms of ETS and DpR but the major difference of disease control seems to be mediated by rapidly developing treatment resistance. From a biological perspective, the hypothesis of secondary resistance is supported by previously described mechanisms of resistance against *BRAF-*inhibitor based therapy in *BRAF* mutant mCRC (Ahronian et al. 2015; Corcoran et al. 2012; Hazar-Rethinam et al. 2018; Prahallad et al. 2012). From a clinical point of view, the idea of secondary resistance is supported by a pooled analysis of several clinical trials that reports on the poor prognosis in patients with *BRAF* mutant mCRC after failure to first-line therapy and the consecutive challenge to ensure the transfer to second-line therapy (Seligmann et al. 2017).

Due to the small number of patients analyzed and the intensive treatment regimens applied in all patients, it is impossible to determine whether the unfavorable tumor control after DpR is specific for *BRAF*-MT mCRC in general or for *BRAF*-MT mCRC treated with FOLFOXIRI regimens. If the intensity of antitumor treatment plays a role in the evolution of mCRC—based on our observations—it should be questioned if the clinical challenge of treating *BRAF*-MT mCRC is best addressed by maximizing initial efficacy or by applying a sequential therapy that might induce less tumor response but is possibly associated with less aggressive tumor evolution in the course of disease. This perspective is supported by somehow inconsistent observations on *BRAF*-MT mCRC patients in current phase III trials (Cremolini et al. 2020; Cremolini et al. 2015b; Modest et al. 2019a). Based on findings from the TRIBE 1/2-trials and the XELAVIRI study, it could be hypothesized that clear evidence for treatment escalation in *BRAF* mutant mCRC is still lacking and less intensity is not necessarily associated with worse outcome.

Similar analyses from the BEACON-trial investigating a targeted combination therapy with BRAF-, MEK-, and EGFR inhibition in *BRAF*-MT mCRC patients could clarify to which extent tumor dynamics observed in the VOLFI trial are also evident with targeted tumor therapy (Kopetz et al. 2019).

Interestingly, no difference in the patterns of disease progression was observed in the VOLFI trial, likely a consequence of the small number of patients analyzed. Future studies should evaluate if disease progression, especially progression including new lesions (as correlate of aggressive tumor evolution and dismal prognosis), depends on molecular or clinical subgroups (Giessen et al. 2013; Modest et al. 2017).

The presented results are limited due to the retrospective nature of the analysis and the use of novel endpoints. Furthermore, there were a limited number of patients in the analyzed subgroups precluding definite conclusions. The hypotheses generated in this manuscript should, therefore, be evaluated in larger patient cohorts.

## Conclusion

BRAF-MT patients and patients with right-sided primary mCRC treated within the VOLFI-trial derive a less profound response to treatment as compared to BRAF-WT patients and patients with left-sided primary tumor. In particular, our observations hint towards an aggressive tumor evolution in patients with BRAF-MT tumors, which may be molecularly detectable at the time of DpR. These findings theoretically challenge the currently practiced aggressive treatment strategy of FOLFOXIRI-based first-line regimens as they may stimulate treatment resistance. We suggest that close monitoring of BRAF-MT patients may include the continuous monitoring of clonal evolution of the disease.
